# The Influence of pH on the Emulsification Properties of Heated Whey Protein–Pectin Complexes

**DOI:** 10.3390/foods13142295

**Published:** 2024-07-21

**Authors:** Yeyang Wang, Bongkosh Vardhanabhuti

**Affiliations:** Division of Food, Nutrition, and Exercise Sciences, University of Missouri, Columbia, MO 65211, USA

**Keywords:** whey protein, pectin, emulsion, protein–polysaccharide complex, emulsification properties

## Abstract

Interactions between proteins and polysaccharides could improve protein functional properties. Most studies focus on the formation of complex coacervates at pHs < pI. Much less attention has been given to the interactions at pHs > pI, especially when the mixtures are heated. The objective of this study was to investigate the emulsification properties of heated whey protein isolate (WPI) and pectin complexes formed at near neutral pHs. Heated soluble complexes (Cpxs) were formed by heating mixed WPI (3 wt% protein) and pectin (0 to 0.60 wt%) at pH 6.0, 6.5, or 7.0 at 85 °C for 30 min. Emulsions (5 wt% oil, 0.5 wt% protein, and pH 5.5) were characterized by measuring droplet size, zeta potential, rheological properties, and creaming stability. The results showed that, regardless of heating pH, Cpxs formed more stable emulsions with significantly smaller droplet sizes, higher negative charges, and less shear-thinning behavior in comparison to emulsions stabilized by heated WPI (*p* < 0.05). At fixed pectin concentrations, the emulsions stabilized by Cpx formed at pH 7.0 were the most stable. Increasing pectin concentrations led to a decrease in mean droplet sizes and an increase in negative charge. Maximum stability was achieved with the emulsion stabilized by Cpx formed with 0.60 wt% pectin at pH 7.0. The formation of Cpxs under proper conditions will allow for the utilization of WPI in a wider range of applications and fulfill the consumer need for clean label food products.

## 1. Introduction

Whey protein is the major co-product of fermented dairy products. It is widely used for a variety of applications in the food industry. It could be used to alter non-protein ingredients, enhance functional properties, and provide additional nutrients to a food product. However, other components and processing procedures have a huge impact on the stability of whey proteins and influence the quality of food products [1]. To improve the functionality of whey proteins, several modification methods were proposed. One of them is forming complexes with polysaccharides through non-covalent interactions.

Protein–polysaccharide interactions have been a subject of investigation since the early 20th century [2]. Due to their polyelectrolyte nature, proteins and anionic polysaccharides readily associate via electrostatic interactions to form complexes. Complexation between proteins and polysaccharides may result in an improvement in their functional properties compared to the individual macromolecules, including thermal stability [3,4], solubility, emulsification properties [5], and foaming properties [6]. The nature and strength of the interactions strongly depend on molecular properties; the biopolymers’ concentration, pH, and ionic strength [7,8,9,10]. Most studies have focused on protein–polysaccharides complexes or coacervates formed at pHs lower than pI [8,11,12,13], where proteins carry positive charges and can form strong electrostatic interactions with anionic polysaccharides. However, recent studies have shown that complexation still occurs at pHs above pI (e.g., pH 5.2 to 7.0 for WPI), where interactions can exist between chain segments of anionic polysaccharides and the positively charged residue (−${\mathrm{NH}}_{3}^{+}$) groups on the protein [14,15]. These interactions can be enhanced by heating in conditions where the mixed biopolymer solution eventually forms soluble aggregates [15,16].

Emulsion is one of the most commonly used systems in food products. It is relatively unstable due to energetically unfavorable contact between oil droplets and water [17]. The formation of kinetically stable emulsions can be achieved by adding emulsifiers such as small-molecular-weight surfactants and proteins. Due to its high surface activity, whey protein isolate (WPI) can rapidly absorb onto oil droplet surfaces, lower the surface tension, and prevent the droplets from aggregating [18]. However, the emulsification properties of whey protein are highly influenced by pH, ionic strength, and temperature [14,18].

Studies found that complexation between proteins and polysaccharides significantly improved the protein’s emulsification properties compared to single molecules [19,20,21]. Emulsions stabilized by complexes have smaller droplet sizes, a higher surface charge, and higher viscosity compared to those stabilized by a protein [14,22]. Since the interaction between two biopolymers is mainly driven by electrostatic interactions, the emulsification properties of the complexes are influenced by pH, the salt environment, and the nature of the biopolymers [5,14,22,23,24]. Polysaccharides can adsorb onto oil droplets primarily emulsified with proteins through electrostatic interactions and increase the thickness and the charge potential of the interfacial layer between the oil droplets. Subsequently, the steric and electrostatic repulsions between the oil droplets are enhanced, leading to improved emulsion stability [5]. The majority of studies that report the emulsification properties of protein–polysaccharide complexes mostly focus on the complexes formed at pHs < pI [25,26] and only a limited number of studies focus on the emulsification properties of heated soluble complexes formed at neutral or near neutral pHs.

The objective of this study was to investigate the emulsification properties of heated soluble complexes formed by heating WPI and low-methoxyl pectin at pHs higher than pI. Their emulsification properties were evaluated at pH 5.5, where the emulsification and stabilizing properties of protein may be diminished. The effects of pectin concentration and heating pH on the emulsification properties and stability of the emulsions were determined.

## 2. Materials and Methods

### 2.1. Material

Whey protein isolate (BiPro) was kindly provided by Davisco Foods International Inc. (Le Sueur, MN, USA). According to the manufacturer, the powdered WPI contained 97.9 wt% protein and 2.1 wt% ash and 0.3 wt% fat (dry weight basis) and 4.7 wt% moisture. Pectin (LM-12 CG) was kindly donated by CP Kelco Inc. (Atlanta, GA, USA). Commercial soybean oil (Great Value) was obtained from the local grocery store. All chemicals used were of analytical grade.

### 2.2. Sample Preparation

WPI stock solutions (10 wt% protein) and pectin stock solutions (2 wt%) were prepared by slowly dissolving the protein or polysaccharide powder in appropriate amount of deionized (DI) water (>17 MΩ). The WPI and pectin solution were stirred for 2 h at room temperature and at 65 ℃. Both stock solutions were left overnight in the refrigerator (4 ℃) for complete hydration. On the next day, the solutions were warmed to room temperature for 2 h.

#### 2.2.1. Formation of Heated WPI-Pectin Complex

Stock solutions of WPI and pectin were mixed in appropriate amounts and their pH was adjusted to 7.0, 6.5, or 6.0 using 1 N HCl. DI water was added to reach a final protein concentration of 3.0 wt% and a final pectin concentration of 0 to 0.60 wt%. The mixtures were gently stirred at room temperature for 2 h before being heated in a temperature-controlled water bath at 85 ℃ for 30 min and cooled. Samples were kept in at 4 ℃ for 18 h.

#### 2.2.2. Emulsion Preparation

All emulsions (5 wt% oil, 0.5 wt% protein, and 0–0.10 wt% pectin at pH 5.5) were obtained by a two-step process of emulsification [14]. Coarse emulsions were prepared by blending oil and aqueous solution together using a laboratory homogenizer, Ultra Turrax T-25 (IKA Works, Wilmington, NC, USA), at 12,000 rpm for 15 s at room temperature. Final emulsion samples were obtained using an ultrasonic processor Sonics VC 505 (Sonics & Materials, Inc., Newtown, CT, USA, power 500 W, frequency 24 kHz) with a sonotrode (6 mm, approx. length 142 mm, titanium) for 3 min (40% amplitude of maximum power). Sodium azide (0.02 wt%) was added as an antimicrobial agent. After emulsification, the emulsions were slowly acidified to pH 5.5. Then, the emulsions were stirred for 1 h before final sonication for 30 s using similar conditions as those described above.

### 2.3. Particle Size and ξ-Potential Measurement

The particle size distribution and ξ-potential of aqueous solutions and emulsions were determined with a Zetasizer Nano ZS (Malvern Instruments Ltd., Worcestershire, UK) equipped with a 633 nm laser and 173° detection optics at 25 ℃. Each aqueous solution was diluted to 0.3 wt% protein with 5 mM phosphate buffer at its respective pH. Emulsion samples were diluted in a 1:100 ratio with 5 mM phosphate buffer at pH 5.5 [14]. All measurements were carried out in triplicate.

### 2.4. Rheological Properties’ Measurement

The rheological properties of the emulsions were determined with a Kinexus Pro Rheometer (Malvern Instruments Ltd., Worcestershire, UK) equipped with a cone (40 mm diameter, 4° angle) and plate geometry [14]. Fresh emulsion samples were loaded on the lower plate and the upper cone geometry was gently lowered to a gap of 0.05 mm. A solvent trap setting was used to prevent evaporation. The flow behavior of the sample was obtained under a shear rate ramp, from 0.1 s^−1^ to 200 s^−1^, at 25 °C. The flow behavior index and consistency coefficient were calculated using the Power Law model, as shown in Equation (1), where σ and $\dot{\gamma }$ are the shear stress and shear rate, and n and *K* are the flow behavior index and consistency coefficient, respectively. Each treatment was measured in triplicate.

(1) $$\sigma =K{\dot{\gamma }}^{n}$$

### 2.5. Creaming Index Measurement

Fresh emulsion samples (10 mL) were pipetted into a cylindrical glass tube (internal diameter = 16 mm, height = 100 mm). Subsequently, the tubes were sealed with Parafilm M film (Pechiney Plastic Packaging Company, Chicago, IL, USA) to prevent evaporation. Emulsion samples were stored at ambient temperature (~22 °C) for 15 days. Emulsion stability was determined by measuring the height of a distinctive clear or semi-transparent bottom serum phase layer on day 5, 10, and 15 after emulsion preparation. The extent of creaming was characterized by the creaming index (CI, %) = (HS/HI) × 100%, where HS is the height of the serum layer and HI is the initial height of the emulsion. Creaming measurements were conducted in triplicate.

### 2.6. Statistical Analysis

Minitab (Minitab Inc., State College, PA, USA, version 18) was used to analyze significant differences (*p* < 0.05) between treatments by one-way ANOVA. The comparisons between the mean values were evaluated by the Tukey HSD test.

## 3. Results and Discussion

### 3.1. Particle Sizes and Surface Charge of Cpxs

**Figure 1:**
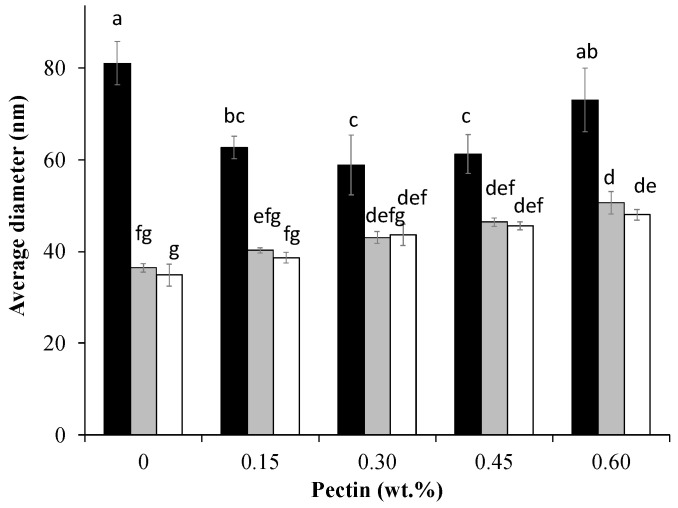
Mean particle sizes of complexes formed at different pectin concentrations and at pH 6.0 (black), pH 6.5 (gray), and pH 7.0 (white). Different letters indicate significant differences (*p* < 0.05) between samples.

**Figure 2:**
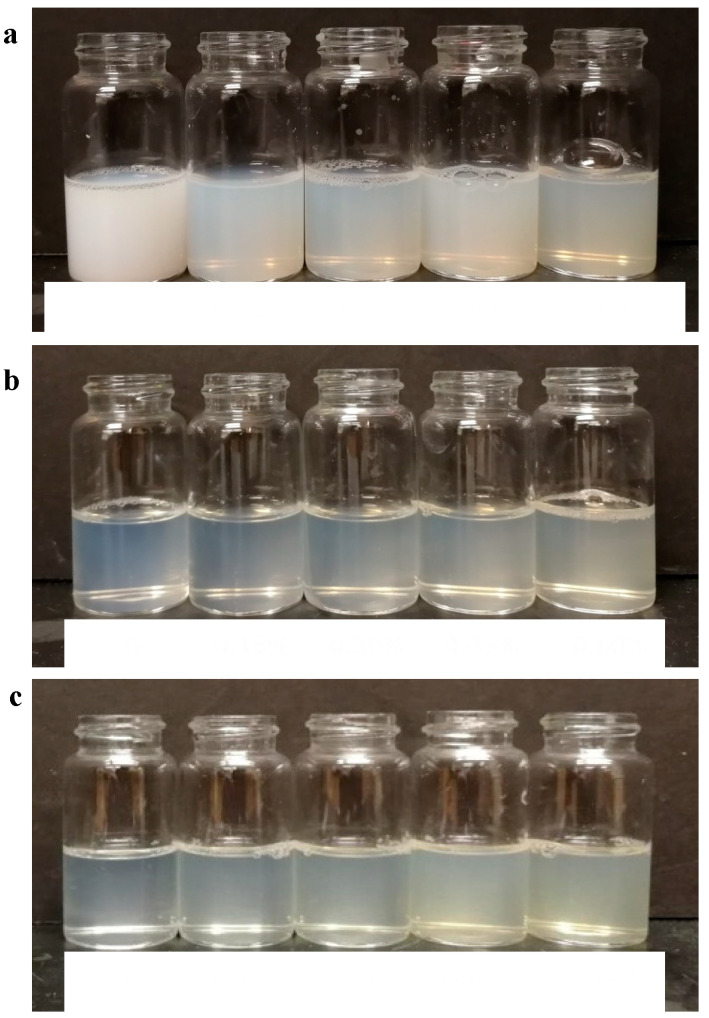
Effect of heating, pH, and pectin concentration on the aggregation of WPI (3 wt%) heated at pH 6 (**a**), pH 6.5 (**b**), and pH 7 (**c**). From left to right (0, 0.15, 0.30, 0.45, and 0.60 wt% pectin).

The effects of heating pH and pectin concentration on the formation of heated WPI–pectin complexes (Cpxs) were determined by measuring their aggregates’ size and surface charge. The Z-average mean diameter of the Cpx solutions is shown in Figure 1. Similar to what was reported by Zhang et al. [4], the mean diameters of the unheated WPI and pectin were 5 nm and 700 nm, respectively. Mean particle sizes significantly increased after heating due to the formation of heated soluble aggregates. The mechanism of whey protein’s aggregation has been well established [27,28,29]. During heating, the folded hydrophobic groups and thiol groups become exposed and interact with other protein molecules through disulfide bonds, non-covalent interactions, and hydrophobic interactions [30]. Protein heated at pH 6.0 showed significantly larger particle sizes than those heated at pH 6.5 and pH 7.0. A study found that different mechanisms are involved in whey protein aggregation below and above pH 6.5 [31]. At neutral pHs, the disulfide bond is highly responsible for the formation of large aggregates, whereas non-covalent interactions are more prominent at lower pHs [32]. The opaque color (Figure 2) and size data at pH 6.0 indicate the formation of large aggregates. This is likely due to the relatively weak repulsion between protein molecules which cannot prevent the self-association of protein.

When WPI and pectin were heated together, the particle size distribution showed a single peak in all treatments, whereas peaks of pectin and protein disappeared, indicating that heated soluble complexes were formed between the two biopolymers. An example is shown in Supplementary Figure S1 for the Cpx formed by heating WPI and 0.15% pectin at pH 7.0. Previous studies have demonstrated that soluble complexes could be formed at neutral pHs [15,16,21]. At pHs > pI (e.g., 5.2–7.0), an interaction still occurs between the chain segments of anionic polysaccharides and the positively charged residue (−${\mathrm{NH}}_{3}^{+}$) groups on the protein. At pH 6, the z-average decreased from 81.1 ± 4.7 nm to 62.7 ± 2.5 nm when heated with 0.15 wt% pectin. Complexation with pectin can suppress the interactions between protein molecules by providing an additional electrostatic repulsive force [4,33,34]. The addition of 0.30 wt% pectin led to a further decrease in size to a minimum of 59.0 ± 6.5 nm. When the pectin concentration exceeded 0.45 wt%, the average aggregate size started increasing again, indicating that phase separation became more dominant. The effect of pectin on protein aggregate sizes showed different patterns at pH 6.5 and 7.0. The increasing pectin concentration led to increased mean particle sizes when mixed biopolymers were heated at pH 6.5 or 7.0. At higher pH values, the repulsion between protein molecules was enhanced with additional pectin, resulting in microphase separation and larger aggregates [15]. It should be noted that, despite differences in mean diameters, the z-average values ranged from 34.6 to 81 nm. Solutions became opaque after heating at pH 6.0, whereas those heated at pH 6.5 and 7.0 remained translucent. The difference in appearance suggests that different shaped of aggregates were formed [35]. Aggregates that are large and more spherical appear opaque, while those with more linear shapes appear more clear or translucent [36,37].

**Figure 3:**
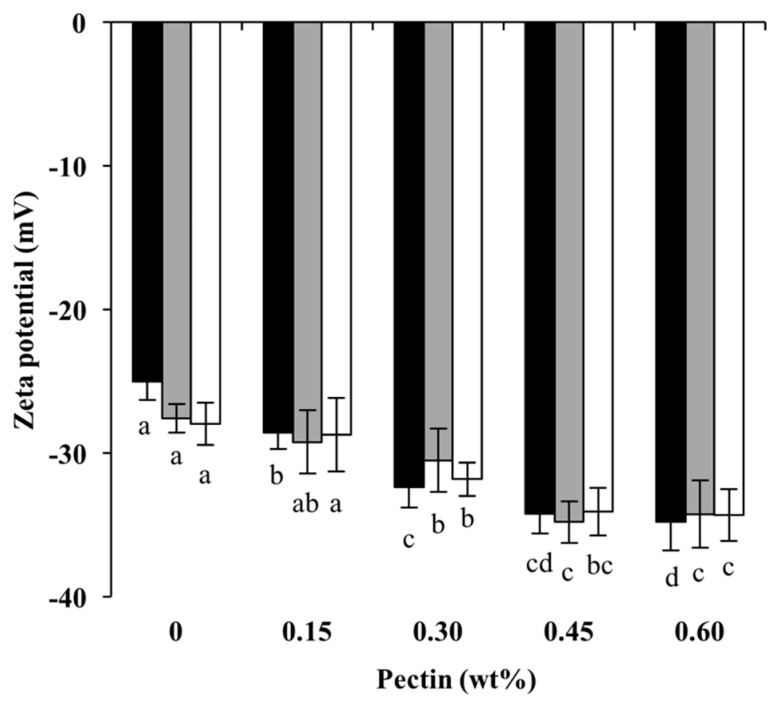
Zeta potentials of Cpxs formed at pH 6.0 (black), 6.5 (gray), and 7.0 (white) and different pectin concentrations. Different letters indicate significant differences (*p* < 0.05) between samples at the same heating pH.

Since the main interaction between whey protein and pectin is electrostatic, a change in surface charge density is an ideal tool to investigate the interaction between the two biopolymers. The ξ-potentials of Cpxs formed at different pHs and pectin concentrations are shown in Figure 3. All heated WPI carried a net negative charge at pHs above pI and became more negative with increasing pHs, from −25.1 ± 1.2 mV at pH 6.0 to −27.9 ± 1.5 mV at pH 7.0. At all pH values, the ξ-potentials became more negative as the pectin concentration increased, indicating an existing interaction between protein and pectin. At high pectin concentrations, e.g., 0.45 wt% to 0.60 wt%, there is no significant difference in ξ-potential (e.g., plateau around −34 mV), indicating that the proteins were fully covered by pectin molecules. A similar trend was reported for a heated WPI–pectin complex at pH 4.75 and 6.0–7.0 [15,34] and a whey protein–xanthan gum conjugate formed at pH 7 [38]. It is notable that the effect of pH on the change in surface charge was not as prominent as pectin concentration. Interestingly, similar ξ-potentials were observed among different pHs at the same pectin concentration (*p* > 0.05). It is possible that the interaction between the protein and pectin was still relatively limited at high pH values and that more interactions were allowed at a lower pH. Salminen and Weiss also reported a similar surface charge for a heated whey protein–pectin complex formed at pH 6.0–7.0 [39]. The particle size and ξ-potential results in this study demonstrate the interactions between protein and pectin through thermal treatment at neutral pHs. Pectin concentration and pH have different effects on particle size and surface charge.

### 3.2. Characterization of o/w Emulsions

#### 3.2.1. Surface Charge

Heated WPI or heated complex (Cpx) solutions were used as the aqueous phase during emulsion formation. The final emulsions contained 5.0 wt% oil and 0.5 wt% protein and were at pH 5.5. The final concentration of pectin in the emulsions was 0, 0.025, 0.050, 0.075, or 0.10 wt%, which corresponded to 0, 0.15, 0.30, 0.45, or 0.60 wt% pectin, respectively, in the Cpx solutions.

**Figure 4:**
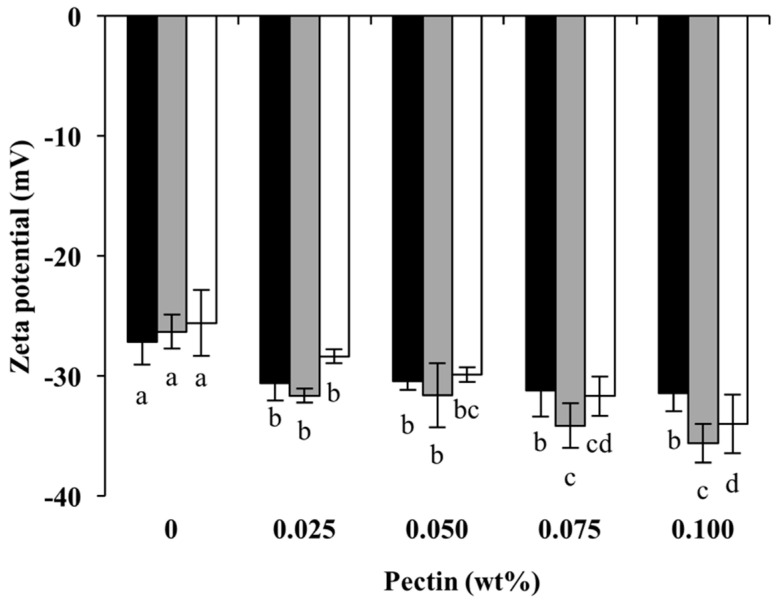
Zeta potentials of emulsions stabilized by Cpxs formed at pH 6.0 (black), heated at pH 6.5 (gray), heated at pH 7.0 (white) and with different pectin concentrations. Different letters indicate significant differences (*p* < 0.05) between samples at the same heating pH.

The influences of the formation pH and pectin concentration on the surface charge of the fresh emulsions are shown in Figure 4. In the absence of pectin, the net charge of the emulsion droplets remained negative at pH 5.5, from −27.1 ± 2.0 mV to −25.6 ± 2.8 mV. Generally, an absolute ζ-potential of at least 30 mV is required to form a stable dispersion. The incorporation of pectin caused a noticeable change in ζ-potential. For emulsions prepared with Cpxs formed at pH 6.0, the surface charge changed from −27.1 ± 2.0 mV to −30.6 ± 1.5 mV with the presence of 0.025 wt% pectin. Anionic groups of pectin were bound with cationic patches on the protein through electrostatic interaction, increasing the negative charges on the droplet surface. However, the ζ-potential appeared to be constant despite the further addition of pectin, suggesting that the absorption of Cpxs or pectin onto the droplet surface was saturated at low pectin concentrations [14]. A similar trend has been reported with emulsions stabilized by 0.5 wt% β-lactoglobulin with 0–0.15 wt% pectin at pH 3 and 5 [23]. The high repulsion of the droplets may prevent the additional adsorption of pectin or Cpxs at the interface [25]. Interestingly, the saturated pectin concentrations were at 0.075 wt% and 0.10 wt% for pH 7.0 and 6.5, respectively. Aggregate size and the amount of soluble aggregates vs. native protein could play a role in the absorption of pectin to protein at the interface. A study found that the heating pH significantly affected yield of soluble aggregates from pH 6 to 7 [40]. At 0.075% pectin, the ζ-potentials of all emulsions were <−30 mV, indicating colloidal stability.

#### 3.2.2. Droplet Size

**Figure 5:**
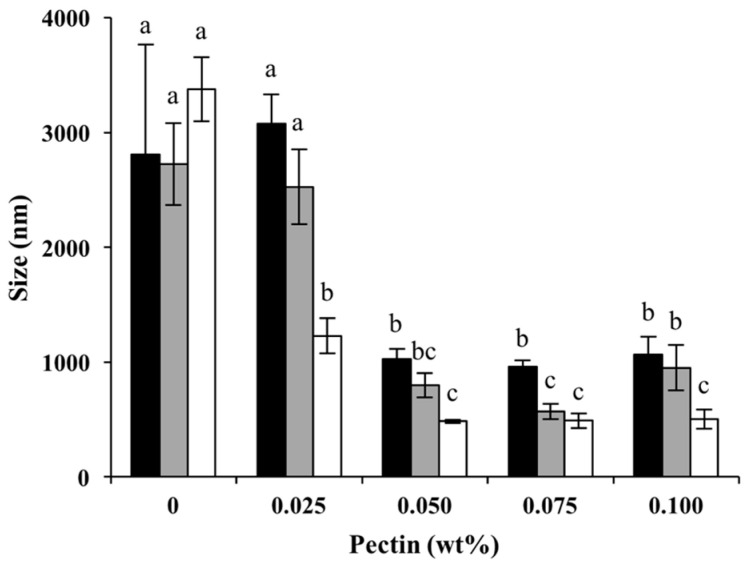
Mean particle sizes of emulsions stabilized by Cpxs formed at pH 6.0 (black), heated at pH 6.5 (gray), heated at pH 7.0 (white) and with different pectin concentrations. Different letters indicate significant differences (*p* < 0.05) between samples at the same heating pH.

The combined influence of pectin concentration and heating pH on the mean diameter of the droplets is shown in Figure 5. In the absence of pectin, the mean diameter of the droplets ranged from 2.80 ± 0.95 μm when the WPI was heated at pH 6.0 to 3.37 ± 0.28 μm when the WPI was heated at pH 7.0. Since the final pH of the emulsions was close to the isoelectric point, the lack of charge repulsion at the interface can promote the droplets’ aggregation [41]. The low electrostatic repulsion between the oil droplets at pHs near pI was too small to prevent flocculation [5,14,17]. Aggregation and flocculation during emulsion preparation could also result in large polydispersity, as shown by a large standard deviation. The addition of pectin even at 0.025 wt% significantly decreased the droplets’ sizes within Cpxs at pH 7.0 due to the additional electric repulsion and steric hindrance from adsorbed pectin. With increasing pectin concentrations, the mean droplet sizes continued to decrease, indicating a lower degree of flocculation and coalescence due to higher negative charges and steric hindrance from the pectin [10,13,34,42]. Emulsions with smaller oil droplet sizes tend to be more stable and more visually appealing, provide better texture and mouthfeel, and have enhanced bioavailability in the delivery of nutrients and bioactive compounds [26,43].

The influence of heating on emulsion formation was also investigated. The mean droplet sizes of emulsions stabilized by heated protein showed no significant effect (*p* > 0.05) of the heating pH. However, the heating pH appeared to be one of the major factors in the emulsification properties of Cpxs. Emulsions prepared with Cpxs formed at pH 7.0 showed the smallest droplet sizes. A study found that 1 wt% WPIs heated at pH 7 formed fibrillar aggregates, while those heated at pH 6 were mainly compact and spherical. An intermediate mixture of both morphologies was observed at the intermediate pH of 6.6 [40]. Smaller size and a filamentous structure allowed for the faster interfacial adsorption of particles formed at higher pHs, resulting in the formation of oil droplets with smaller sizes [44].

The emulsions formed by emulsifying an oil with protein–polysaccharide complexes made at pHs > pI (e.g., similar to the Cpxs in this study) are called mixed emulsions [45]. The negatively charged Cpxs adsorb at the oil–water interface [45]. Some WPI molecules can remain in the Cpx solutions at low pectin concentrations while the pectin molecules are saturated with protein. No negative charges on pectin are available for bridging to another Cpx-coated oil droplet [45]. This is supported by the droplet size results, which show no change or reduced droplet sizes in emulsions stabilized by the Cpxs compared to the Control (0% pectin).

#### 3.2.3. Rheological Properties of Emulsions

**Figure 6:**
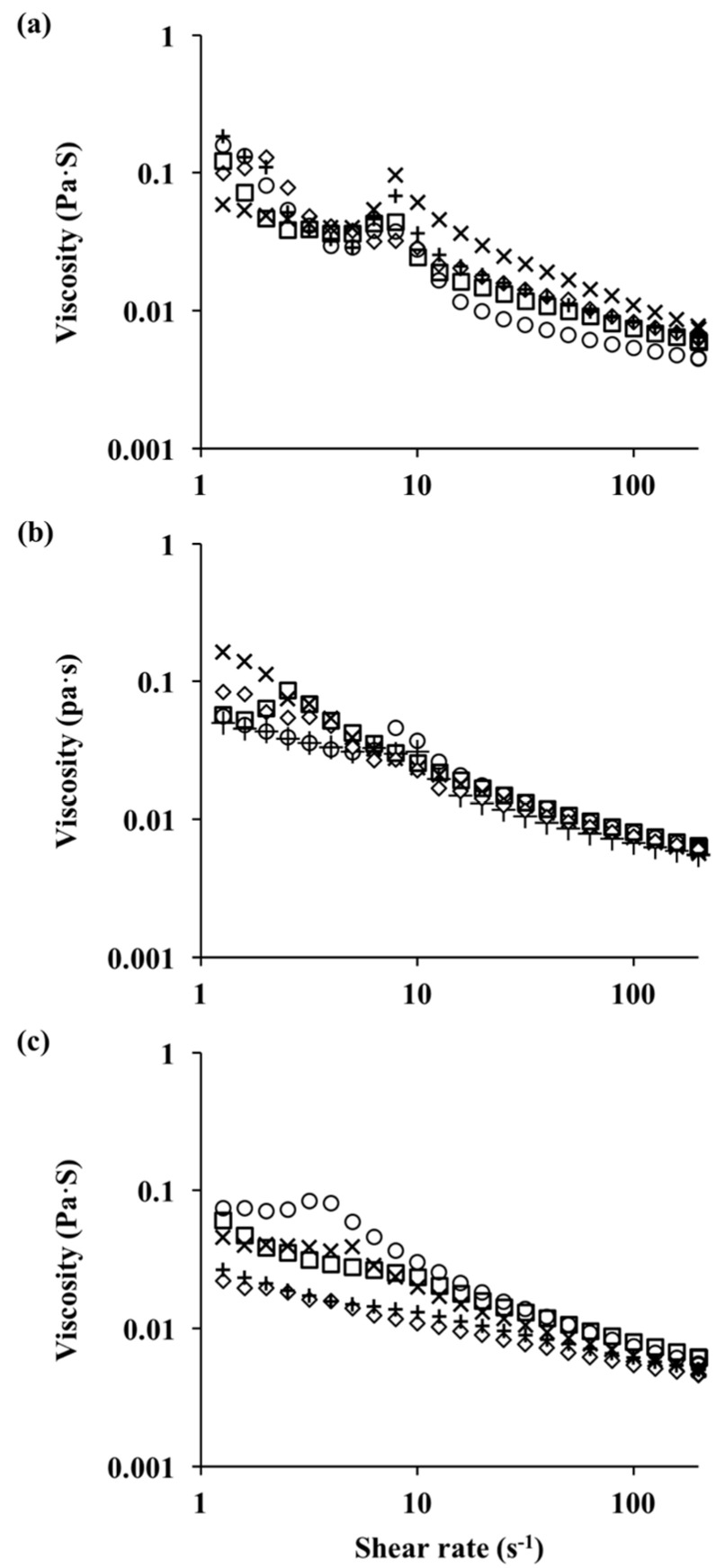
Apparent viscosity of fresh emulsions stabilized by Cpxs formed at pH 6 (**a**), pH 6.5 (**b**), and pH 7 (**c**) with different pectin concentrations; ○ = 0; × = 0.025%; + = 0.050%; ◇ = 0.075%; □ = 0.100%.

The rheological properties were measured immediately after the emulsions’ preparation. The flow behavior curves of fresh emulsions are shown in Figure 6. A Power Law model was applied to describe their rheological properties. The values of their consistency coefficient (K) and flow behavior index (n) are listed in Table 1. The consistency coefficient is a parameter related to viscosity. The flow behavior index indicates the flow behavior of a liquid. Generally, flow behavior is divided into three categories: shear-thinning (n < 1), shear-thickening (n > 1), and Newtonian (n = 1).

In this study, all emulsions showed shear-thinning behavior, with an n less than 1. Their shear-thinning behavior could be due to the flow characteristic of hydrocolloids in the aqueous phase and flocs been gradually broken up under increased shear rates [46]. In the absence of pectin, emulsions stabilized by heated WPI exhibited highly shear-thinning behavior, with n values of 0.127, 0.076, and 0.118 and K values of 0.288, 0.478, and 0.405 at pH 6.0, pH 6.5, and pH 7.0, respectively. These emulsions also showed a deflection point, which might be due to the disruption of flocs, thus reducing their effective volume fraction and viscosity with the increasing shear rate [5,14,47].

The rheological behavior of the fresh emulsions was highly influenced by complex formation. Compared to the pectin concentration, the effect of the heating pH is more pronounced. Generally, emulsions stabilized by Cpxs formed at pH 6.0 or pH 6.5 did not show significant changes in their K or n value. For emulsions with Cpxs formed at pH 7.0, their K values were significantly lower compared to samples without pectin, and the emulsions were also less shear-thinning. At 0.10 wt% pectin, the K value slightly increased and n value slightly decreased, which could be due to the effect of unabsorbed pectin in the aqueous phase [14,48,49]. It should be noted that, at lower pectin concentrations, emulsions stabilized by Cpxs formed at pH 6.0 showed the lowest n and the highest K value (*p* < 0.05), which corresponded to large droplet sizes, as previous shown. The presence of large droplets due to coalescence in the emulsion can cause an increase in viscosity and shear-thinning behavior [50]. At higher pectin concentrations, the mean droplet diameters of emulsions stabilized by Cpxs formed at different pH were similar; however, emulsions formed from Cpxs at pH 7 were less viscous and less shear-thinning compared to those formed at pH 6.0 and 6.5. Since the droplet size measurement was made from diluted emulsions, differences in rheological properties suggest that emulsions stabilized with Cpxs at pH 6.0 and 6.5 could have some degree of flocculation, but flocs dissociated with dilution. Another key advantage of having emulsions with low viscosity is that they can be utilized in beverage applications.

#### 3.2.4. Emulsion Stability

**Figure 7:**
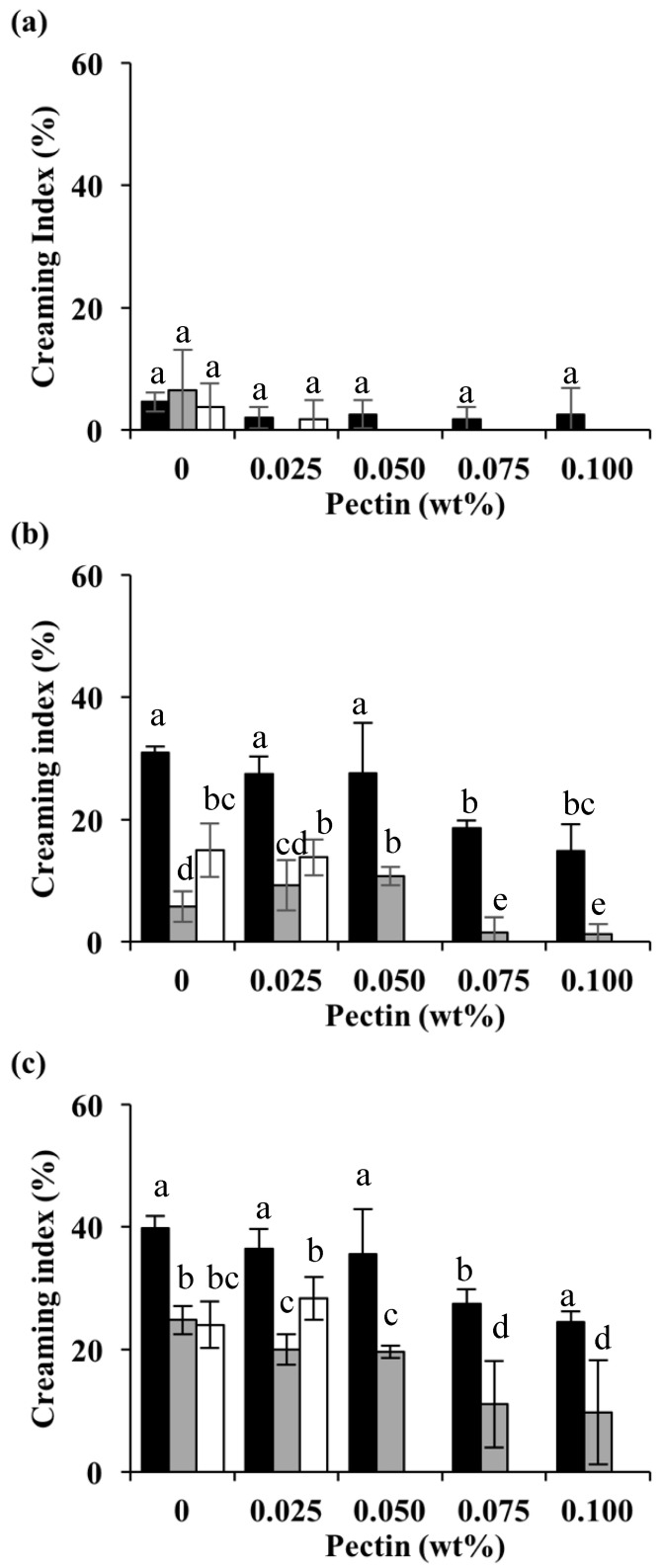
Creaming stability of emulsions prepared with Cpxs formed at pH 6.0 (black bar), pH 6.5 (gray bar), and pH 7.0 (white bar) at different pectin concentrations after day 1 (**a**), day 5 (**b**), and day 15 (**c**). Each value is an average of three replications ± standard deviation. Different letters indicate significant differences (*p* < 0.05) within the same storage period.

**Figure 8:**
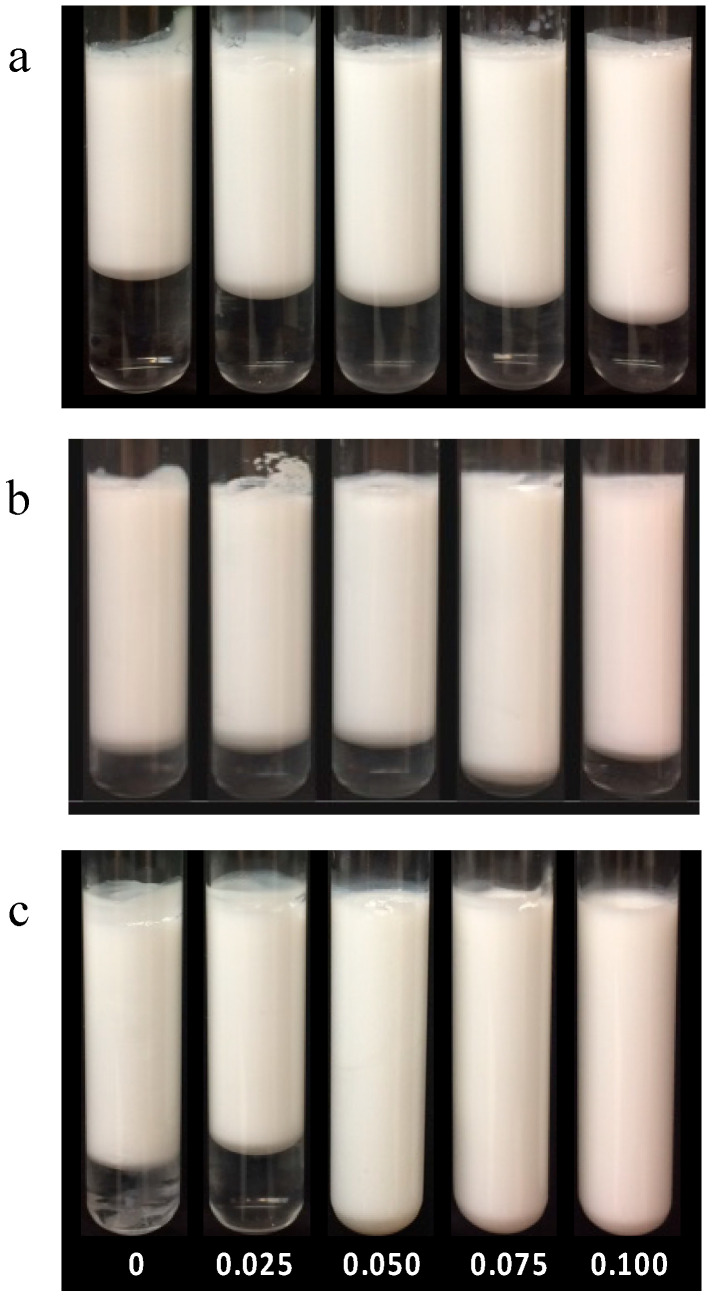
Emulsions (5% oil, 0.5% protein, and pH 5.5) stabilized by heated whey protein or a Cpx after 15 days. Cpxs were formed at pH 6 (**a**), 6.5 (**b**), and 7.0 (**c**). From left to right, the final emulsions contained 0, 0.025, 0.050, 0.075, or 0.1% pectin.

Whey proteins have been widely used as an emulsifier in the food industry. Similar to other proteins, WPI-stabilized emulsions are sensitive to pH and lose their stability at pHs close to their pI (~5.2) [41]. Figure 7 shows the effect of pectin concentration and Cpx formation pH on emulsion stability against creaming during 15 days of storage. Photos of the emulsions after 15-day storage are shown in Figure 8. It should be noted that the emulsion formed with unheated WPI separated into two layers within one day. The separated emulsions showed two layers: an opaque cream layer at the top and a transparent layer at the bottom, suggesting that all of the droplets were aggregated and rapidly moved upwards due to gravity [5]. In the absence of pectin, emulsions stabilized by heated protein showed separation within 1 day. The high viscosity of the emulsions stabilized by heated protein did not lead to improved stability against creaming. These results also support that the high viscosity observed in emulsions stabilized by heated WPI was due to the formation of flocs, as flocculation enhances the creaming rate [51]. Large droplet size and insufficient electrostatic repulsion in the protein-stabilized emulsion caused a high degree of flocculation and coalescence.

Cpxs clearly improved emulsion stability and the effects of heating pH and pectin concentration were prominent. Regardless of the complex formation pH, increasing the pectin concentration led to improved stability. The improvement of creaming stability is in line with smaller droplets and increased negative charges. During emulsification and with a pH adjustment to 5.5, more binding sites on the proteins were available to interact with the pectin. Any free excess pectin in the aqueous phase could interact with protein-covered droplets [26]. At low pectin concentrations, insufficient charge repulsion and steric stabilization could lead to a demixing of the emulsions [52], as demonstrated by a lack of stability against creaming. At high pectin concentrations, the net charge on the Cpx becomes sufficiently large that electrostatic repulsion between the adsorbed Cpx stabilizes the emulsion system. A steric stabilization effect could also play an important role in emulsion stabilization. In addition, cross-linking between the pectin molecules formed a relatively weak gel-like network, increasing the viscosity and enhancing stability, as seen with the emulsions stabilized by Cpxs formed at pH 7.0 [23].

Across the pectin concentrations, emulsions stabilized by Cpxs formed at pH 6.0 were the least stable, and creaming was observed within 24 h even at the highest pectin concentration. Emulsions stabilized by Cpxs formed at pH 7.0 were the most stable. No creaming was observed after the 15-day storage when the emulsions contained at least 0.05 wt% pectin. The effect of the heating pH could be supported by the size and viscosity results. It is likely that structural differences play a role in the complexes’ emulsification and stabilization properties. With a smaller and more fibrillar structure, the Cpx formed at pH 7.0 could lead to the formation of smaller droplets and more stable emulsions. Future work is needed to determine the relationship between the structural, physicochemical, and interfacial properties of the complexes.

The limited emulsifying ability and emulsion stabilization property of proteins at pHs near pI are well recognized. It is essential to use preparation conditions that do not promote droplet flocculation and a combination that provides sufficient electrostatic and/or steric repulsion between the droplets. The ability of the Cpx to form stable emulsions under this condition opens up opportunities for many applications, such as mildly acidic beverages and sauces. In addition, oil-in-water emulsions are often used as delivery systems due to their ability to contain oil-soluble, water-soluble, and amphiphilic functional components in a single system. Future work could determine whether the Cpx can enhance the delivery of bioactive compounds in food emulsions.

## 4. Conclusions

Complexation between the protein and pectin led to changes in the surface charge and structure of the heated aggregates. The resulting heated WPI and pectin complexes exhibited improved emulsification properties at pHs near pI, as shown by their decreased droplet sizes, increased surface charge potential, and increased stability against creaming. Both heating pH and pectin concentration play significant roles in the physical properties and emulsification properties of the complexes. Optimum emulsification properties and maximum emulsion stability were observed when the Cpxs were formed at pH 7.0 and with higher pectin contents. Future work will focus on the interfacial characterization and self-assembly properties of these Cpxs at the oil–water interface.

## Figures and Tables

**Table 1:** Power Law model parameters of emulsions stabilized by Cpxs formed at different pHs and pectin concentrations.

Heating pH	Pectin wt%	K (Pa·s^n^) *	n *	R^2^
6.0	0	0.127 ± 0.03 ^a^	0.288 ± 0.06 ^a^	0.85 ± 0.06
	0.025	0.133 ± 0.01 ^a^	0.378 ± 0.09 ^a^	0.93 ± 0.01
	0.050	0.213 ± 0.03 ^b^	0.262 ± 0.04 ^a^	0.86 ± 0.04
	0.075	0.156 ± 0.02 ^ab^	0.334 ± 0.01 ^a^	0.96 ± 0.01
	0.100	0.146 ± 0.03 ^ab^	0.316 ± 0.03 ^a^	0.91 ± 0.03
6.5	0	0.076 ± 0.01 ^a^	0.478 ± 0.03 ^b^	0.98 ± 0.02
	0.025	0.091 ± 0.03 ^ab^	0.367 ± 0.02 ^ab^	0.96 ± 0.02
	0.050	0.076 ± 0.02 ^a^	0.477 ± 0.06 ^a^	0.98 ± 0.01
	0.075	0.160 ± 0.02 ^b^	0.312 ± 0.06 ^a^	0.89 ± 0.03
	0.100	0.113 ± 0.04 ^ab^	0.391 ± 0.06 ^b^	0.98 ± 0.01
7.0	0	0.118 ± 0.01 ^d^	0.405 ± 0.02 ^a^	0.98 ± 0.03
	0.025	0.053 ± 0.00 ^b^	0.533 ± 0.01 ^b^	0.99 ± 0.01
	0.050	0.027 ± 0.01 ^a^	0.633 ± 0.04 ^b^	0.98 ± 0.00
	0.075	0.029 ± 0.00 ^a^	0.639 ± 0.05 ^b^	0.99 ± 0.00
	0.100	0.082 ± 0.01 ^c^	0.422 ± 0.02 ^a^	0.97 ± 0.00

* Consistency coefficient (K) and flow behavior index (n) were determined by fitting the flow curve to the Power Law model. Each value is an average of three samples ± standard deviation. Different letters indicate significant differences (*p* < 0.05) within the same heating pH.

## Data Availability

The original contributions presented in the study are included in the article/Supplementary Material, further inquiries can be directed to the corresponding author.

## Supplementary Materials

The following supporting information can be downloaded at https://www.mdpi.com/article/10.3390/foods13142295/s1, Figure S1: Particle size distributions of native WPI, pectin and Cpx (3% WPI, 0.15% pectin, pH 7.0).
