# Supplementary figures and images for: The Influence of pH on the Emulsification Properties of Heated Whey Protein–Pectin Complexes
Source: Foods. 2024 Jul 21;13(14):2295. doi: 10.3390/foods13142295 (PMC11275619; doi:10.3390/foods13142295)

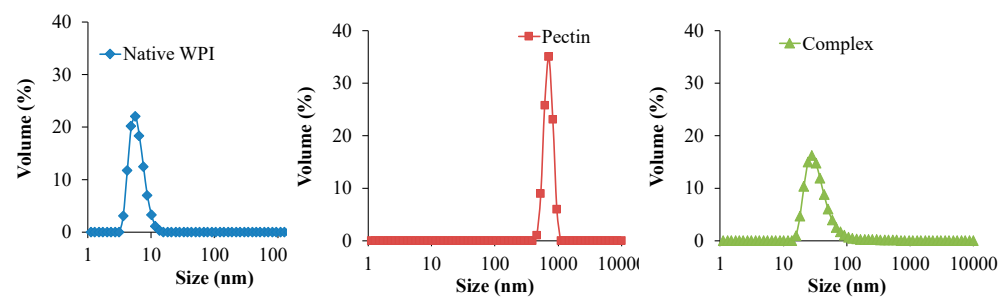

**Figure S1.** Particle size distributions of native WPI, pectin and Cpx (3% WPI, 0.15% pectin, pH 7.0)

Supplement: Supplementary file 1 [file foods-13-02295-s001.zip › foods-3031818-supplementary.pdf]
